# The Effects of Scraping Therapy on Local Temperature and Blood Perfusion Volume in Healthy Subjects

**DOI:** 10.1155/2012/490292

**Published:** 2012-05-14

**Authors:** Qin-Yan Xu, Jin-Sheng Yang, Bing Zhu, Li Yang, Ying-Ying Wang, Xin-Yan Gao

**Affiliations:** Institute of Acupuncture and Moxibustion, China Academy of Chinese Medical Sciences, Beijing 100700, China

## Abstract

*Objective.* We aim to study the therapeutic effects of scraping by investigating the changes of temperature and local blood perfusion volume in healthy subjects after scraping stimulation, and to explore the mechanism of scraping stimulation from the points of microcirculation and energy metabolism. *Methods.* Twenty-three health subjects were included in this study. Local blood perfusion volume and body surface temperature was detected at 5 min before scraping stimulation, 0, 15, 30, 60 and 90 min after scraping using Laser Doppler imager and infrared thermograph. *Results.* Significant increase was noted in the blood perfusion volume in the scraping area within 90 minutes compared to the baseline level and non-scraping area (*P* < 0.001). Compared with non-scraping area, an increase of body temperature with an average of 1°C was observed after scraping stimulation (*P* < 0.01). *Conclusion.* Scraping can significantly improve the blood perfusion volume and increase the temperature in the scraping area, promoting the local blood circulation and energy metabolism.

## 1. Introduction

Scraping, called Gua Sha in Traditional Chinese medicine (TCM), is one of the unique non-medicinal external therapies of TCM under the guidance of the theory of meridians and acupoints. It involves using a smooth-edged instrument for surface frictioning to intentionally raise transitory petechiae and ecchymosis [1, 2]. To date, scraping has shown pain-relieving effects on myalgia and chronic pain [2–5], and can improve blood stasis and inflammation [6]. Although several reports indicated the effects of scraping therapy in clinical and experimental practices, the mechanism is still not clear. Recently, Tian et al. reported blood perfusion volume increased immediately after skin scraping in rabbits using laser Doppler imager [7]. In this study, we aim to determine the changes of the local blood perfusion volume and skin temperature after scraping in healthy subjects.

## 2. Methods and Materials

### 2.1. Laser Doppler Imager

PeriScan PIM II Laser Doppler Perfusion Imager (LDPI; Perimed AB, Jarfalla, Sweden) was used to measure skin perfusion volume. A low power 670 nm wavelength was applied. A medium scanning pattern was used. The image primitive was set as 0.75 mm × 0.75 mm. The image size was set at 40 mm × 40 mm. The apparatus was connected to a PC computer to obtain the blood flow images of the body surface. The laser blood flow image and visual image of the detected areas were measured via LDPI 2.5 Image Software. The blood flow of the body surface was measured by Doppler frequency shifts which is proportional to a blood flow-related variable and is expressed in arbitrary perfusion unit (PU) [8]. The blood perfusion volume and the body position at different time points were analyzed after comparing the laser blood flow images and direct images demonstrated by the Laser Doppler Perfusion Imager.

### 2.2. Infrared Thermograph

WP-1 type of infrared thermograph with a temperature resolution of 0.08°C was applied for thermal images in our study. Based on the infrared radiation photography, the apparatus was connected to a PC computer to convert thermo energy into temperature. The distributions and changes of body temperature were displayed as colorful images. A 3.41 version image processing software was used to analyze the data obtained from the images.

### 2.3. Scraping Stimulation

Scraping stimulation was performed by trained therapists using a buffalo horn scraper and a skin lubricant (Jinlongkang, Beijing Jinlong Kang Er Fu Scraping Cupping Research Institute, Beijing, China) to decrease friction. Scraping was conducted on the erector spinal muscle above the back spine (from C7-T10) along the bladder meridian of the right side. The scraping areas is 6-7 cm in width and 20 cm in length. Infrared thermal images were collected at scraping area from C7 to T7. Laser Doppler images were collected in two areas at the scanning centers of bilateral sides of the back spine (scraping area and non-scraping area at the opposite side) respectively. The areas were 4.5 cm lateral to the spinous process of the 4th thoracic vertebra.

### 2.4. Subjects

Twenty-three healthy subjects (12 males, 11 females) aged from 20 to 40 years old were enrolled after physical examination. Laboratory room temperature were maintained at 24°C−27°C without direct sunlight, infrared radiation, and indoor/outdoor ventilation.

### 2.5. Experimental Procedure

The subjects were seated in a square stool in the laboratory with their back exposed. Before collecting the infrared temperature images, the subjects were needed to stay calm for 15 min to adapt to the room temperature. Infrared temperature images were collected at a sitting position. Then laser Doppler images of both sides of the selected areas at a prone position. After scraping for 5 min, both infrared temperature images and the laser Doppler images of the above areas mentioned were collected immediately after scraping (0 min), 15 min, 30 min, 60 min, and 90 min after scraping respectively.

### 2.6. Data Collection

For infrared thermal images, the subject sat erectly at a distance of 1.5 m to the infrared thermograph. Then the detected area was determined and fixed with a calibration circle. The thermographic imaging system was input into a PC computer to save the infrared images and thermal images. For laser Doppler imaging, the blood perfusion volume of the selected scraping area on the right back and symmetrical non-scraping area on the left side were collected by laser Doppler imager. The images were processed by LDPI 2.5 imaging software for offline analysis.

### 2.7. Statistical Analysis

Data were all presented as mean ± SD. Statistical analysis was performed using SPSS 17.0 Software. A Student's *t* test was performed for the analysis of changes of temperature and blood perfusion volume between pre- and post-scraping, and scraping and non-scraping at different time points. *P* < 0.05 was considered as statistical significance.

## 3. Results

After scraping, all the 23 subjects (100%) reported obviously warm accompanied by slight pain at the scraping area. They all felt relax and comfort after scraping. It was observed that the skin became slightly red, and then subcutaneous hyperaemia and subcutaneous bloody spots were found in the local scraping area.

### 3.1. Changes of Blood Perfusion Volume before and after Scraping

**Figure 1:**
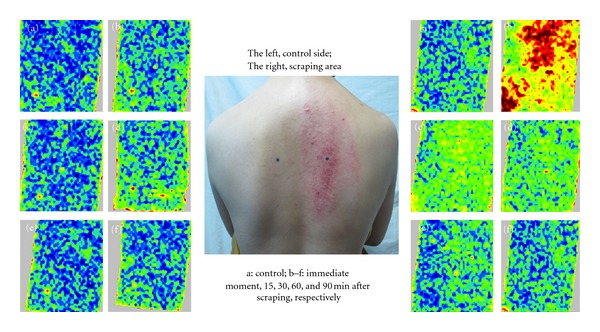
Visual image (middle) taken at 5 min after scraping showed that the skin of the scraping area turned apparently red. Laser Doppler images (left, non-scraping side; right, scraping side) showed the blood perfusion volume. Images (a)–(f) were taken at 5 min before scraping, 0 min, 15 min, 30 min, 60 min and 90 min after scraping stimulation, respectively.

**Figure 2:**
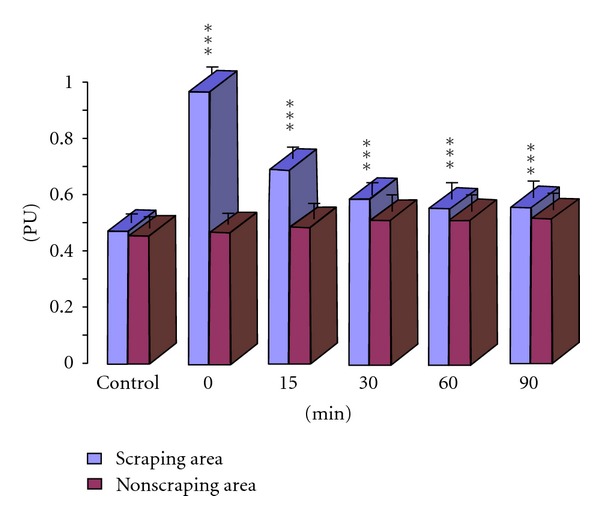
Changes of blood perfusion volume in scraping area and non-scraping area. ***P < 0.001, compared with non-scraping area at the same time point.

Significant increase of blood volume was observed in the scraping area compared with the baseline level. PU values were 1.0-fold higher compared with the baseline level (0.966 ± 0.203 versus 0.469 ± 0.103, Table 1). Significant difference was noted in the blood perfusion volume within 90 minutes after scraping compared with the non-scraping area. (*P* < 0.001; Figures 1 and 2, Table 1).

### 3.2. Changes of the Local Skin Temperature before and after Scrapin

**Figure 3:**
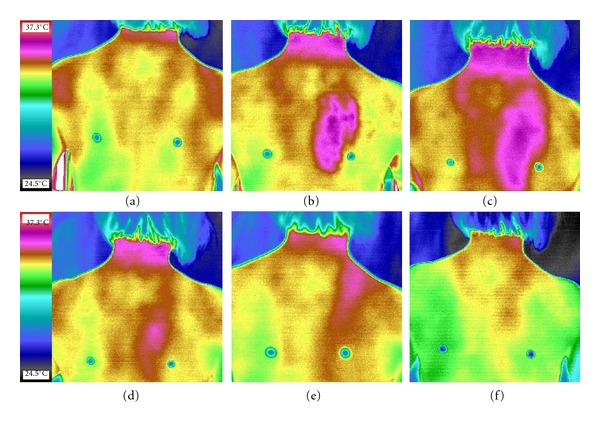
The infrared thermograph images showed the skin temperature of the right body side (scraping) increased significantly after stimulation. Skin temperature increased in the scraping area and extended onto the opposite side and the neck 15 min after scraping. The local temperature increase lasted about 1 hour. (a)–(f): image otabined at 5 min before scraping, 0, 15, 30, 60, and 90 min after scraping.

**Figure 4:**
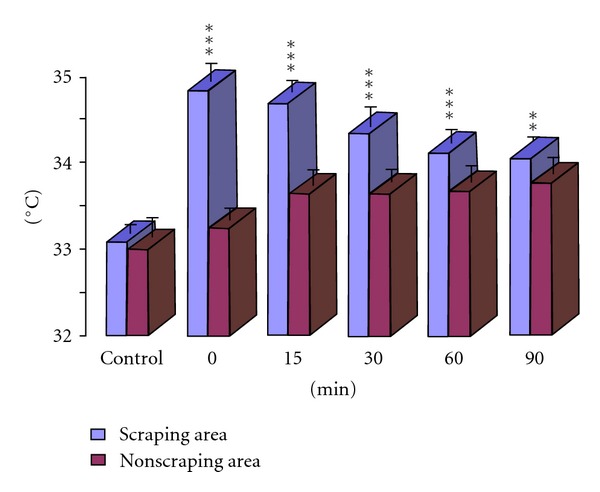
Changes of temperature in the scraping area and non-scraping area. **P < 0.01, ***P < 0.001, compared with non-scraping area at the same time point.

As is shown by infrared thermograph, the skin temperature of the scraping area increased significantly with the average temperature increased more than 1°C. Compared with the skin temperature obtained in the opposite non-scraping area and the scraping area before stimulation, significant increase of skin temperature was observed within 90 minutes after scraping, respectively (*P* < 0.05, Figures 3, and 4, Table 2).

### 3.3. Correlation of Changes of Temperature and Blood Perfusion Volume in the Scraping Area

**Figure 5:**
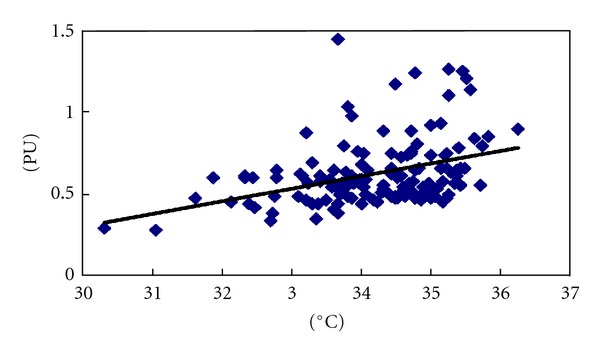
Correlation analysis between temperature and blood perfusion volume in the scraping area.

With regard to the skin temperature and local blood volume obtained within 90 minutes after scraping, a close correlation was noted between skin temperature and the local blood volume in the scraping area (*r* = 0.383, *P* < 0.01, Figure 5). Both temperature and blood flow perfusion values were still higher 90 min after scraping compared with the baseline level (Tables 1, and 2).

## 4. Discussion

 Scraping, called Gua Sha in TCM, is one of the physical stimulating therapies. Previous reports indicated that physical therapies such as acupuncture, moxibustion, massage, scraping and cupping basically shared similarities in their functions and mechanisms as they all developed from external stimulating therapies [9]. In the 56th Chapter of *Plain Questions*, an ancient works in TCM, it mentioned that “the 12 meridians and collaterals distributed in their relevant cutaneous regions”. Zeng (1999) reported that the scraping performed by stimulating the collaterals on the surface of the body was efficient for the treatment of certain diseases. Therefore, the author speculated that the efficiency of scraping therapy is closely related with the function of collaterals [10]. Though several studies reported the effects of scraping therapy in clinical practices [2–5], its mechanism is still not well defined. In this study, Laser Doppler imager and infrared thermograph were used to detect the effects of scraping therapy on local temperature and blood perfusion volume of human body surface. Macroscopic observations and infrared images showed apparent changes of the local skin color and temperature before and after scraping. Furthermore, quantitative analysis indicated scraping could increase the local microcirculation and metabolism of subcutaneous tissues.

Skin, covering the body surface, contains abundant capillaries functioned as the major organ for temperature regulation and body defense. Under normal conditions, the blood volume of microcirculation is in accordance with the metabolism level of the tissues and organs to keep a dynamic balance. The capacity and rate of substance exchange of external and internal capillary mainly depended on the open volume and permeability of the true capillary. The present study showed that the the blood flow volume in the scraping area significantly increased, especially immediately after scraping. The values of the blood flow increased 1.0-fold higher in the scraping area than those of the non-scraping area (Table 1). Our study is in accordance with the previous report which indicated that Gua Sha caused a 4.0-fold increase in microcirculation PUs at the scraping area for the first 7.5 minutes together with a significant increase in surface microcirculation during the entire 25 minutes of the study period following scraping stimulation (*P* < 0.001) [2]. The obvious increase of blood perfusion volume indicated that scraping stimulation could reflexively regulate the sympathetic vasodilator nerves to relax the precapillary sphincter, increase the local volume of blood flow and the amount of the opening capillaries directly, and promote local blood circulation. Scraping stimulation was possible to cause partial subcutaneous bleeding of the capillaries, resulted in hyperaemia or blood stasis [7], which can otherwise promote the metabolism of the tissues and improve local microcirculation [11–14]. According to the infrared thermograph images, a significant increase was noted in the scraping area. As is shown in Table 2, an average of 1°C was noted after scraping stimulation. Under normal conditions, temperatures at both sides of the body back are nearly the same and symmetrical [15]. Our results also indicated that scraping could lead to a long-lasting (60 min) increase of temperature in the adjacent tissues and even further (Figure 3). It could affect these functions of the surrounding tissues. The effects of scraping to an extended area lies in that it causes more vessel dilation and increase of blood flow volume in the adjacent tissues as the cutaneous arteries trunk on the back are interconnected with each other to form a vessel network [16].

Generally, the blood circulations in human body surface were stable. Once the pressure and muscle relaxation of scraping extruded subcutaneous capillary, capillary network reconstruction and expansion was induced, which resulted in changes of cutaneous blood volume and skin temperature [12, 17]. This phenomenon indicated that scraping could change the subcutaneous micro-vascular pressure, leading to vascular dilation and increase of local temperature and the volume of blood flow of the scraping area. Previous study showed that heat could increase the temperature of the tissues, dilate the capillaries, increase local blood circulation, promote blood and oxygen supply, and strengthen the metabolism of the local tissues [13]. Based on our results, a strict correlation was detected between the blood perfusion volume and skin temperature (*r* = 0.383, *P* < 0.01, Figure 5)

Scraping is performed according to the location of acupuncture points along meridians [18]. According to the previous report, thermal conductivity along meridians and beneath tissues was more remarkable than other parts of the body [19]. In addition, a positive correlation between the therapeutic effects and microcirculatory changes of the suffered areas or relevant points was found [20]. Moreover, a remarkable increase was noted in microcirculation and blood perfusion volume after scraping stimulation in the meridian and points [21]. Our study indicated that the responsive areas of scraping extended to the bladder meridian on both sides of the back spine. embodied by mainly by capillary dilation, obvious temperature change and expanded blood perfusion volume of the scraping areas. Generally, scraping of a tolerable intensity is a positive stimulation on the skin, and can helps to increase the metabolism of the local and adjacent tissues as well as activate physiological functions of the body. The increased temperature and microcirculation could reversely remove the microcirculatory obstruction, especially for arteriole angiectasis and spasm [22]. Scraping, stain stimulation mode, could change the skin color of the local scraped area and produce warming or even slightly pain. A variety of scraping stimulation performed on body surface would help to relieve the muscular spasm and improve the local metabolism of tissues, reduce the tension of blood vessels and nerves, and eliminate or reduce the negative impact of somatic disorders on visceral functions [23]. Therefore, it is an effective way for removing the microcirculatory obstruction.

In our study, Laser Doppler and infrared thermal imaging techniques were used for the first time for the detection of the skin temperature and blood volume in healthy subjects. The effect of scraping therapy was analyzed to clarify the mechanism of scraping from microcirculation and energy metabolism. Our study provided theoretical and clinical guidances on the research of meridians and collaterals for further studies. Further studies about the effects of the different scraping techniques on pressure changes of subcutaneous microcirculatory system, and the influences of scraping stimulation on meridians and collaterals should be performed in the near future.

## Figures and Tables

**Table 1:** Blood perfusion volume at different time points in scraping area and non-scraping area (PU), (*n* = 23, $\overset{̅}{x}\pm SD$).

PU	Scraping area (right)	Non scraping area (left)	*P* values
Before scraping	0.469 ± 0.103	0.453 ± 0.105	*P* > 0.05
immediately	0.966 ± 0.203	0.465 ± 0.089	*P* < 0.001
15 min	0.685 ± 0.158	0.483 ± 0.076	*P* < 0.001
30 min	0.586 ± 0.075	0.510 ± 0.080	*P* < 0.001
60 min	0.553 ± 0.064	0.504 ± 0.061	*P* < 0.001
90 min	0.558 ± 0.066	0.514 ± 0.052	*P* < 0.001

**Table 2:** Mean infrared temperature at different time points in the scraping area and non-scraping area (*n* = 23, $\overset{̅}{x}\pm SD$), **P < 0.01.

(°C)	Scraping area (right)	Non scraping area (left)	*P* values
Before scraping	33.057 ± 1.116	32.989 ± 1.137	*P* > 0.05
Immediately	34.837 ± 0.743	33.233 ± 0.851	*P* < 0.001
15 min	34.703 ± 0.614	33.633 ± 0.673	*P* < 0.001
30 min	34.343 ± 0.855	33.640 ± 0.733	*P* < 0.001
60 min	34.123 ± 0.769	33.688 ± 0.674	*P* < 0.001
90 min	34.065 ± 0.838	33.771 ± 0.69	*P* < 0.01
